# TRANSANAL HAEMORRHOIDAL DEARTERIALIZATION WITH MUCOPEXY (THD-M) FOR TREATMENT OF HEMORRHOIDS: IS IT APPLICABLE IN ALL GRADES? BRAZILIAN MULTICENTER STUDY

**DOI:** 10.1590/0102-672020190001e1504

**Published:** 2020-08-24

**Authors:** Carlos Walter SOBRADO, Sidney KLAJNER, José Américo Bacchi HORA, Anderson MELLO, Fabricio Marcondes Luciano da SILVA, Marcos Onofre FRUGIS, Lucas Faraco SOBRADO

**Affiliations:** 1 Department of Gastroenterology, Faculty of Medicine, University of São Paulo, SP, Brazil; 2 Hospital 9 de Julho, São Paulo, SP, Brazil; 3 Albert Einstein Hospital, São Paulo, SP, Brazil; 4 Dona Helena Hospital, Joinville, SC, Brazil; 5 Military Hospital, Porto Alegre, RS, Brazil

**Keywords:** Hemorrhoids, Transanal hemorrhoidal dearterialization, Doppler-guided, Mucopexy, Complications, Hemorroidas, Desarterialização hemorroidária transanal, Doppler, Mucopexia, Complicações

## Abstract

**Background:**

Transanal haemorrhoidal dearterialization with mucopexy (THD-M) is a valuable option for treating patients with haemorrhoidal disease. However, there is still controversy with regard to its efficacy for more advanced grades.

**Aim:**

To evaluate the effectiveness of THD-M technique for treating hemorrhoidal disease and to compare the immediate and late results in different grades.

**Method:**

Seven hundred and five consecutive patients with Goligher’s grade II, III or IV symptomatic haemorrhoids underwent surgical treatment using the THD-M method in five participating centres. Six well-trained and experienced surgeons operated on the patients. Average follow-up was 21 months (12-48).

**Results:**

Intraoperative complications were observed in 1.1% of cases, including four cases of haematoma, two of laceration of the mucosa, and two of bleeding. All of these were controlled by means of haemostatic suturing. In relation to postoperative complications, the most common of these were as follows: transitory tenesmus (21.4%); pain (7.2%); mucosal or haemorrhoidal prolapse (6.4%); residual skin tag (5.6%); faecal impaction (3.2%); haemorrhoidal thrombosis (2.8%); bleeding (2.1%); anal fissure (0.7%); and anal abscess (0.3%). Most of the complications were treated conservatively, and only 7.5% (53/705) required some type of surgical approach. There was no mortality or any severe complications. The recurrence of prolapse and bleeding was greater in patients with grade IV haemorrhoidal disease than in those with grade III and II (26.54% and 7.96% vs. 2.31% and 0.92% vs. 2.5% and 1.25%), respectively.

**Conclusion:**

The THD-M method is safe and effective for haemorrhoidal disease grades II and III with low rates of surgical complications. However, for grade IV hemorrhoids, it is associated with higher recurrence of prolapse and bleeding. So, THD-M method should not be considered as an effective option for the treatment of grade IV hemorrhoids.

## RESUMO

**Racional:**

A desarterialização hemorroidária transanal associada à mucopexia (THD-M) tem sido indicada como alternativa à hemorroidectomia convencional para o tratamento da doença hemorroidária nos seus variados graus. No entanto, ainda hoje existe controvérsia com relação a sua eficácia para os graus mais avançados.

**Objetivo:**

Avaliar a eficácia da técnica THD-M para tratamento doença hemorroidária e comparar os resultados imediatos e tardios nos diferentes graus.

**Método:**

Entre julho de 2010 e setembro de 2015, 705 pacientes consecutivos com hemorroidas sintomáticas de graus II, III e IV foram submetidos ao tratamento cirúrgico pelo método THD-M e acompanhados por um período médio de 21 meses (12-48). As operações foram realizadas por seis cirurgiões com experiência em cirurgia colorretal, em três estados brasileiros.

**Resultados:**

Complicações intraoperatórias foram observadas em 1,1% dos casos, incluindo quatro casos de hematoma, dois de laceração da mucosa e dois de sangramento. Todos foram controlados com sutura hemostática. As complicações pós-operatórias mais comuns foram: tenesmo transitório (21,4%); dor (7,2%); prolapso mucoso ou hemorroidário (6,4%); plicoma residual (5,6%); impactação fecal (3,2%); trombose hemorroidária (2,8%); sangramento (2,1%); fissura anal (0,7%) e abscesso anal (0,3%). Não houve casos de complicações severas ou mortalidade perioperatória. A recorrência do prolapso e sangramento foi maior na doença hemorroidária grau IV do que nos graus III e II (26.54% e 7.96% vs. 2.31% e 0.92% vs. 2.5% e 1.25%; p<0,001), respectivamente.

**Conclusão:**

O método THD-M é seguro e efetivo no tratamento da doença hemorroidária nos graus II e III com baixo índice de complicações. No entanto, para a doença hemorroidária de grau IV essa técnica está relacionada com maior índice de recorrência e complicações tardias, não devendo ser considerada opção eficiente neste estágio de doença.

## INTRODUCTION

Hemorrhoidal disease (HD) is the most common anal condition seen within coloproctology, and it affects one third of young adults in developed countries^14^.

Although excisional surgical methods (including conventional open and closed haemorrhoidectomy) are effective over the long term and have low recurrence rates, they are often accompanied by intense postoperative pain, and may involve complications such as residual fissures, anal incontinence and anal stenosis. Furthermore, it takes a long time for these patients to return to their habitual activities, and thus, there is a significant social and economic impact^5^ ^,^ ^17^.

More recently, minimally invasive techniques such as mechanical anopexy (stapled haemorrhoidopexy) and Doppler-guided haemorrhoidal arterial dearterialization (transanal haemorrhoidaldearterialization, or THD) have been described. Because these techniques do not involve making incisions in the anoderm, there is less postoperative pain than in conventional haemorrhoidectomy^8^ ^,^ ^19^ ^,^ ^20^.

The “procedure for prolapsed haemorrhoids” (PPH) was introduced by Longo^18^ in 1998 and consists of resection of a circular band of rectal mucosa (mucosectomy) and fixation of its distal part inside the canal above the pectinate line, with the aid of a stapler. Initial studies with short-term follow-ups showed that this method was less painful than haemorrhoidectomy and that it also enabled an earlier return to habitual activities^18^ ^,^ ^33^.

Studies of the PPH technique utilizing larger samples and longer-term follow-up have shown that the recurrence rates for prolapse and bleeding are higher and that there is a risk of severe complications, including chronic anorectal pain, rectovaginal fistula, rectal perforation, pelvic sepsis and even death^7^ ^,^ ^21^.

In 1995, Morinaga *et al.* ^*20*^ described a new technique for treating symptomatic internal haemorrhoids with dearterialization, which they named haemorrhoidal artery ligation (HAL or THD). This consisted of ligature of the terminal haemorrhoidal branches, which had previously been identified with the aid of a Doppler probe coupled to an anoscope. The main aim of the procedure was to ligate these branches, which would diminish the arterial hyperflow in the anal vascular cushions, thereby controlling the main symptoms of the haemorrhoids, i.e., prolapse and bleeding. In 2007 Dal Monte *et al.* ^*1*^ published an article in which they added mucopexy to ligation of the haemorrhoidal artery branches for patients with prolapsing haemorrhoids of grades III and IV, with the aim of treating the haemorrhoidal mucosal prolapse. Addition of mucopexy in the cases of patients who presented prolapsing haemorrhoids can restore anal anatomy and physiology^6^ ^,^ ^27^. Over the past decade, surgical equipment has improved and many articles have been published, showing that this method including dearterialization and mucopexy is safe and efficient for treating haemorrhoidal disease, with low morbidity^6^ ^,^ ^11^ ^,^ ^25^ ^,^ ^28^ ^,^ ^31^.

This multicentre observational study had the objective to evaluate the effectiveness of theTHD-M method in the treatment of symptomatic haemorrhoids in varying grades, with regard to control of both prolapse and bleeding, and to analyze the postoperative morbidity.

## METHODS

Between July 2010 and September 2015, 705 consecutive patients (457 males, 66.4%; 248 females, 32.6%) with a mean age of 43 years (range: 18 to 85) with Goligher’s grade II, III or IV symptomatic haemorrhoids underwent surgical treatment using the THD Doppler method in five participating centres. Six well-trained and experienced surgeons operated on the patients. All surgeons who participated in this study already had experience with anorectal operations and had previously performed more than ten THD-mucopexy (THD-M) procedures, before starting this study. The patients were followed up over a mean period of 21 months (12 to 48). Each patient’s preoperative evaluation consisted of a proctologic examination, including digital rectal examination and anoscopy, and an assessment of the severity of hemorrhoidal disease according to the classification system proposed by Goligher. A complete medical and surgical history, with an emphasis on aspects relating to haemorrhoidal disease, was gathered. Colonoscopy was performed as a screening measure, according to the patients’ individual risk of cancer, such as a family history of cancer or colorectal adenomas, and age greater than 50 years. Grade II patients were treated with the THD Doppler procedure when more conservative therapies (such as diet, pharmacological therapy, sclerotherapy or elastic ligature) had failed. 

The exclusion criteria included: coagulation defects, pregnancy, active inflammatory bowel disease, previous major rectal surgery, full-thickness rectal prolapse, and colorectal and anal carcinoma. All patients received detailed explanations regarding the THD procedure and signed an informed consent statement in order for the operation to be performed. 

### Operative technique

The procedures were performed under general or spinal anaesthesia, depending on the preferences of the team and the patient. The choice of anaesthetic agents was left to the anaesthetist’s discretion. Antibiotics were not used routinely and there was no preoperative intestinal preparation or enema. If necessary, the anal canal and distal rectum were cleaned after the patient was anaesthetized. All the patients were operated upon in the lithotomy position. The THD procedure was performed using the devices of the THD Doppler device kit (THD S.p.A., Correggio, Italy). This contains a transparent anoscope equipped with a Doppler probe on one of its lateral walls and a light source. The anoscope was fully introduced into the anus until the lower rectum was reached; at this level, six terminal branches of the superior haemorrhoidal artery were generally detected by the Doppler, usually in positions corresponding to one, three, five, seven, nine and eleven o’clock, around the rectal circumference at a distance of four centimetres from the pectinate line. Then the anoscope was moved down slowly until the Doppler probe received a second stronger loud sound. When only dearterialization was planned (in patients without prolapse), all six terminal branches were then ligated approximately 3-4 cm above the pectinate line, at the location where the sound was most audible, using absorbable 2-0 polyglactin thread inserted in a short needle of circumference 5/8 provided as part of the THD kit (THD S.p.A., Correggio, Italy). The arterial ligature involved transfixation of the rectal mucosa and submucosa using “X” format stitches in order to seal the branches of the superior rectal artery. In cases with prolapse, at the level of the strongest Doppler signal, a ”marker point” was placed on the rectal mucosa using electrocauterization. Thereafter, the anoscope was fully reintroduced, and a continuous suturing was performed with four to six stitches, including the marker point and encompassing the haemorrhoidal cushions, taking care to ensure that the most distal stitch was 5 to 10 mm above the pectinate line. Lastly, the thread was knotted at the level of the cranial stitch to lift and fix the mucosal prolapse. A small gel foam roll (from the THD kit) covered with anaesthetic ointment was placed in the anal canal. The aim of mucopexy was to more firmly adhere the mucosa to the deeper layers of the rectal wall, and as a result of the resultant fibrosis, to correct the prolapse. Concomitant procedures such as excision of skin tags, polyps, hypertrophic papilla and fissurectomy were performed according to individual needs. 

### Follow-up

The patients were released from hospital within 24 h, generally after presenting bowel movements. During the postoperative period, the patients were advised to avoid physical exertion and straining during evacuation for two weeks. A liquid-rich diet with fibre supplements and faecal emollients was prescribed. Patients who did not present evacuation within 48 h were instructed to use osmotic laxatives (lactulose ormacrogol 3350) and, if this did not have any effect, to return for a medical reassessment. Analgesia comprising 200 mg of ketoprofen per day, divided into two doses, and 750 mg of acetaminophen three times a day or 1000 mg of dipyrone four times a day, was administered over a period of 5-7 days.

After release from hospital, the patients were assessed one, three and twelve weeks and 12 months afterwards, and then annually or when necessary. The patients were examined and questioned about any symptoms of haemorrhoidal disease (bleeding, prolapse and pain)and about their intestinal habits. Any early complications (up to the 7^th^ postoperative day) or late complications were also reported. Any recurrence of the haemorrhoidal symptoms or persistence of any other symptoms at the end of follow-up was also reported.

### Statistical analysis

Statistical analysis was performed with the objective of making comparisons between the grades of haemorrhoidal disease (grades II, III and IV) and the incidence of complications (recurrence of prolapse or bleeding, and thrombosis) that resulted from using the THD-M technique. To make comparisons between the grades, Fisher’s exact test with a significance level of 1% for two-tailed tests was used, i.e., a test that was more rigorous than the significance level of 5%. The analyses were performed using SPSS software for Windows, version 17 (SPSS, Chicago, IL, USA.

## RESULTS

Table 1 reports clinical characteristics of the 705 patients included in the study. Among them, 22.6% (160/705) were classified as grade II, 61.4% (432/705) as grade III and 16% (113/705) as grade IV. Symptoms reported most often were bleeding (97.2%), prolapse (89.5%), anal discomfort (21.1%), mucorrhea (17.5%) and pruritus (13.5%). The duration of the surgical procedure ranged from 22 to 60 minutes, with a mean of 29 minutes. The length of hospital stay was one day in 84.6% of the cases (n=597), two days in 9.4% of the cases (66 patients), and more than two days in 6% (n=42) of the cases. An additional concomitant procedure was performed on 130 out of the 705 patients (18.4%), including: excision of a plicoma (skin tag), anal fissurectomy with internal sphincterotomy, fistulectomy, excision of a sebaceous cyst, transanal excision of a rectal polyp and excision of a hypertrophic papilla.

**TABLE 1:** Patients’ characteristics

Number of patients (n)	705
Gender	Male 457 (66.4%); Female 248 (33.6%)
Age	Mean: 43 years-old (18-85)
Grade of hemorrhoidal disease	II: 160 (22.6%); III: 432 (61.4%); IV: 113 (16%)
Symptoms	Bleeding 97.2%; prolapse 89.5%; anal discomfort 21.1%; mucorrhea 17.5%; pruritus 13.5%.

There was neither mortality nor any life-threatening morbidity. Intraoperative complications were observed in eight patients (1.1%): four cases of haematoma, two cases of mucosal lacerations, and two cases of bleeding. All of these were treated by haemostatic suturing, using absorbable thread (2-0 polyglactin, Ethicon). During the postoperative period, the main early complications (occurring within the first seven days) were: transitory tenesmus in 21.4% (151/705), which generally improved by the 7^th^ day; difficult-to-treat pain in 7.2% (51/705), which necessitated analgesia greater than what is routinely prescribed; urinary retention in 2.4% (17/705) with the need for bladder catheterization; and post-dural puncture headache in 0.3% (2/705), which was treated conservatively. Faecal impaction occurred in 3.2% (23/705) and was treated by oral osmotic laxatives (lactulose ormacrogol 3350) and/or evacuation enemas.

Beyond the first seven days and during the follow-up period, which ranged from 12 to 48 months (mean of 21 months), the following late complications were observed: recurrence of the prolapse in 6.4% of the cases (44/705), which was treated using an elastic ligature in 15 patients and with a surgical procedure in 29 patients(THD in two; haemorrhoidectomy in 27); residual plicoma (skin tag) in 39 patients (5.6%), among whom 15 underwent surgical resection (2.2%); external haemorrhoidal thrombosis, diagnosed in 2.8% of patients (20/705), but requiring a surgical excision in only seven patients, while the other 13 were managed with analgesics, non-steroidal anti-inflammatory drugs and phlebotonics. Bleeding necessitating surgical revision occurred in 2.1% of the cases (15/705), and haemostatic suturing using absorbable thread (2-0 polyglactin, Ethicon) was performed successfully in all cases. Anal fissure occurred in 0.7% of patients (5/705), and was managed using conservative measures. Two patients (0.3%) underwent drainage of an anal abscess, with a good outcome.

Regarding the overall incidence of late complications and their relationship to the severity of hemorrhoidal disease, the patients with grade IV haemorrhoidal disease were found to achieve poorer control over bleeding and prolapse (Table2).

**TABLE 2:** Late complications vs. degree of haemorrhoidal disease

Late complications	Grade II	Grade III	Grade IV	Total
Recurrence of prolapse	4/160 (2.50%)	10/432 (2.31%)	30/113 (26.51%)	6.4% (44/705)
Recurrence of bleeding	2/160 (1.25%)	4/432 (0.92%)	9/113 (7.96%)	2.1% (15/705)
Thrombosis	2/160 (1.25%)	6/432 (1.39%)	12/113 (10.61%)	2.8% (20/705)

Comparing the results of late complications related to the degree of haemorrhoidal disease (separated into two groups: one including both II and III grades, the second including only IV), statistically significant differences were noted: all of them were higher in the grade IV group (Table 3).

**TABLE 3:** Late complications (Grades II + III vs. Grade IV)

Late complications	Grades II+III	Grade IV	p
Recurrence of prolapse	2.36% (14/592)	26.54% (30/113)	<0.0001
Recurrence of bleeding	1.01% (6/592)	7.96% (9/113)	0.0001
Thrombosis	1.35% (8/592)	10.61% (12/113)	<0.0001

In general, the complications (including both early and late) were mostly low in complexity and managed conservatively (Table 4).There was a need for surgical treatment of some type of complication in 7.5% of the cases (53/705), and all these operations were of low complexity, without mortality.

**TABLE 4:** General incidence of postoperative complications (early and late)

Early complications	% (N)
Tenesmus	21.4% (151/705)
Pain	7.2% (51/705)
Faecal impaction	3,2% (23/705)
Urinary Retention	2.4% (17/705)
Headache	0.3% (2/705)
Late complications	% (N)
Mucosal/hemorrhoidal prolapse	6.4% (44/705)
Residual plicoma (skin tag)	5.6% (39/705)
Hemorrhoidal thrombosis	2.8% (20/705)
Bleeding	2.1% (15/705)
Anal fissure	0.7% (5/705)
Anal abscess	0.3% (2/705)

## DISCUSSION

Treatment of symptomatic haemorrhoids using a Doppler-guided dearterialization technique on the terminal haemorrhoidal branches was proposed by Morinaga *et al.* ^*20*^ in 1995, who named it haemorrhoidal artery ligation (HAL). They performed this technique on 116 patients and reported good results regarding pain resolution and control over prolapsed (78%) and bleeding (95%), despite their short follow-up. With the aim of optimizing the results from the THD technique, some technical modifications have subsequently been introduced, such as performing mucopexy in association with THD^6^ and making changes to surgical materials^25^ ^,^ ^26^.

In Brazil, experience with the THD-M procedure began in July of 2010. In the present study on 705 consecutive patients, the vast majority (84%) presented grade II and III haemorrhoids. At the time when surgery was indicated for the individuals with grade IV haemorrhoidal disease, the possibility of excising the external component concomitantly with THD-M was previously discussed. There was a need to perform an additional surgical procedure in 130 patients (18.4%), 103(14.3%) of whom presented with grade IV haemorrhoidal disease. Careful attention to the following important technical measures is needed: precise identification of the locations of the haemorrhoidal terminal arterial branches using Doppler; firm and precise ligatures, both at the time of dearterialization and when performing anorectal repair (lifting); and implementation of haemostasis when necessary, always by means of suturing. Through these measures, intraoperative complications were observed in only eight patients (1.1%) of our series, all successfully treated. In a multicentre trial published by Ratto*et al.* ^*28*^ intraoperative complications were observed in 0.5% (4/803) of the cases, all similarly managed with good results.

Bleeding subsequent to THD was infrequent and was observed in only 2.1% (15/705) of our patients. This diagnosis was made within the first two weeks after the operation in 12 cases. In three of them, the cause was an ischaemic ulcer; in the remainder, it was due to tearing of the mucopexy suture. Bleeding occurred more frequently among the individuals with grade IV haemorrhoidal disease (Tables 2 and 3). Using theTHD-M procedure, Dal Monte *et al.* ^*1*^ observed postoperative bleeding in 2.1% of their patients (7/330) and Ratto*et al.* ^23^ reported that 1.2% of their 170 patients required surgical haemostasis during the postoperative period. In a multicentre study of 803 patients in Italy who underwent the THD-M technique, 18 (2.2%) presented postoperative bleeding, all of them within the first 30 days. Only seven of them (0.9%) required revision surgery^28^. Zampieri*et al.* ^*35*^ did not observe any bleeding that would necessitate a surgical approach in any of their 46 patients. A Korean group found that 8.2% (8/97) of their patients presented bleeding after undergoing THD-M, and these cases were treated by means of surgical haemostasis, with good results^13^. Loganathan*et al.* ^*16*^ reported that bleeding occurred in 7% (6/85) of their patients, among whom 50% had been using anticoagulants or had received antiplatelet agents before the operation; all of them were successfully treated through conservative measures alone. Care is needed with patients who require an anorectal operation and who have been using coumarins, platelet antiaggregants or any other drug with an effect on blood coagulation.

The main complaints reported by our patients during the early postoperative period were tenesmus, anal pain or discomfort and urine retention. Tenesmus occurred in 21.4% (151/705), is generally transitory within the first two postoperative weeks. The inflammatory process (triggered by the various mucopexy sutures in the low rectum and the consequent local ischaemia and edema) may explain both tenesmus and local discomfort^28^ ^,^ ^30^. The greater the number of running sutures involving the anal canal mucosa and submucosa, the greater the feeling of tenesmus and pain, and the higher the possibility of ischaemia will be^30^. During the mucopexy procedure, the longitudinal sutures should be performed separately from each other, thus preserving a band of normal mucosa between them, with the aim of ensuring adequate arterial irrigation of the low rectum and anal canal, preventing ischaemia and external haemorrhoidal thrombosis. Oral and topical analgesics, non-steroidal anti-inflammatory drugs and phlebotonics, and measures for correcting intestinal constipation and preventing faecalomas (laxative diets and osmotic laxatives), are generally used to control this symptom. Ratto*et al.* ^*23*^ reported that tenesmus occurred in 24.1% (41/170) of their patients and that it spontaneously resolved by the 10^th^ day after the operation. Jeong *et al.* ^13^ reported occurrences of transitory tenesmus in 19.5% (19/97) of their patients.

Urinary retention with the need for bladder catheterization was observed in 2.4% (17/705) of our patients, 14 (2%) of them over the age of 50 years. In the literature, the incidence of this postoperative event following anorectal operations is 4-10% and may arise through several factors: dysfunction of the detrusor muscle of the bladder in response to pain; anaesthesia through spinal block; prostatism; administration of large volumes of fluids after the operation.^.^ Urinary retention rates specifically following the THD method were reported to be 2% by Loganathan *et al.* ^*16*^ *,* 4% by Giordano *et al.* ^*8*^ *,* 0.6%by Dal Monte*et al.* ^1^ (2/330) and8.6% by Ratto *et al.* ^28^

Pain and discomfort, which occur frequently after conventional haemorrhoidectomy, are another constant concern in relation to surgical treatment of haemorrhoidal disease^5^. Most studies have shown that the incidence of pain during the postoperative period following the THD procedure ranges from 1-17%. Thus, this method is less painful than other operative methods described previously (conventional haemorrhoidectomy, PPH and methods using sealants such as Ligasure*)* ^*3*^ ^*,*^ ^*31*^ ^*,*^ ^*35*^ *.* In our series, pain that required use of analgesic (opioids) other that ketoprofen and paracetamol or dipyrone was present in 7.2% (51/705). A systematic review of 17 studies by Giordano *et al.* ^8^ in 2009 (evaluating1996 patients who underwent the THD method) found that the mean rate of postoperative pain was 4.7%. Dal Monte *et al.* ^1^ reported that 2.7% of their 330 patients had high-intensity pain (VAS>8). An Australian group reported that pain necessitating opioid medication was presented by 16% of their patients^16^. Ratto*et al.* ^23^ reported that anorectal pain was present in 27 (15.9%) of their patients during the postoperative period, of whom 15 reported pain after defecation, 12 reported pain during evacuation, and four reported pain with no relationship with evacuation. However, only eight patients (4.7%) required major analgesia for duration of 6.6±4.1 days. Pain is generally present in cases involving complications such as thrombosis, abscess or anal fissure, or when the continuous line of suturing of the mucopexy affects the pectinate line^13^. 

Recurrence of hemorrhoidal prolapse after dearterialization with mucopexy was observed in 6.4% (44/705) of our patients. Thirty had grade IV haemorrhoidal disease (26.54% - 30/113), grade III (2.31% - 10/432) and 2.50%, 4/160 (1.4%) had grade II. In the group including both grade II and III haemorrhoidal disease, recurrence of prolapse occurred in 2.36% (14/592), while it was seen in 26.54% (30/113) of patients with grade IV disease (p<0.0001). In situations involving either small or single prolapses, which are the most common situation, rubber band ligation, in association with correction of constipation, is usually effective. In cases involving either major or circumferential prolapse, surgical treatment should be indicated and reconstruction of the mucopexy or excisional haemorrhoidectomy is the technique indicated^27^.

Among the 44 patients (6.4%) with recurrence of prolapse in our study, 15 were treated with rubber band ligation and 29 with surgery, with a good outcome. Two patients were treated with a repeated THD procedure and 27 with haemorrhoidectomy using the closed technique, with excision of the residual piles and skin tag. Dal Monte *et al* ^1^ compared individuals with grades III and IV haemorrhoidal disease who underwent haemorrhoidal dearterialization alone, with those who underwent this in association with mucopexy, and observed haemorrhoidal prolapse recurrence rates of 6% vs. 3.7% in the groups with grade III haemorrhoidal disease and 50% vs. 11.1% in the groups with grade IV haemorrhoidal disease respectively. They concluded that although mucopexy presented better results with regard to controlling prolapses, their sample was not large enough to reach statistical significance. In a systematic review published in 2009, including 1996 patients who underwent the dearterialization technique without mucopexy, the mean recurrence rate for haemorrhoidal prolapse was 9% (range: 0 to 37%)^8^. Using the THD plus mucopexy technique, Ratto*et al.* ^23^ reported that haemorrhoidal prolapse recurred in 7.6% of their patients and mucosal-hemorrhoidal prolapse recurred in five patients (2.9%) and that seven patients (4.1%) required surgical reintervention: four received a new THD procedure and three haemorrhoidectomy. Faucheron*et al.* ^4^ reported that prolapse recurrence occurred in 9% of their 100 patients with grade IV haemorrhoidal disease who underwent THD-M procedure, after a mean follow-up of 34 months. Treatments for recurrence included a new THD procedure in three cases, haemorrhoidectomy in three cases and conservative management in the remaining three. In 2014, Giordano *et al.* ^*10*^ performed haemorrhoidal dearterialization and mucopexy on individuals with grade IV haemorrhoidal disease and, after a mean follow-up period of 32 months, observed the presence of tenesmus in 10% of the patients and recurrence of the prolapse in 3%. The initial studies using the dearterialization method alone on individuals with voluminous grade IV haemorrhoidal disease produced discouraging results because of the difficulty in correcting the prolapse^6^ ^,^ ^32^. Subsequently, several other studies using an combination of mucopexy with dearterialization have been published, showing better control over the prolapse, with haemorrhoidal prolapse recurrence rates ranging from 3-17%, with the highest rates having been seen in grade IV haemorrhoidal disease and after follow-up periods longer than 12 months^12^ ^,^ ^29^ ^,^ ^32^.

In comparison with other techniques, the recurrence rate after THD-M seems to be similar to the rate after PPH^9,12,31^ and equal to or slightly greater than the excisional methods^2^ ^,^ ^3^ ^,^ ^32^, but THD has the advantage because of lower postoperative pain, reduced risk of serious complications (incontinence or stenosis) and earlier return to routine activities^2^ ^,^ ^9^.

As observed in our study, other series published about the THD technique have also reported greater recurrence rate in grade IV patients and have recommended discussing this association with patients before the procedure is performed^8^ ^,^ ^22^ ^,^ ^34^.

It is important to emphasize from our study that the THD with mucopexy method was shown to be safe. Among 53 patients who underwent reoperation, all were subjected to low-complexity procedures and neither severe sequelae nor mortality were observed. We did not observe any patients with permanent anal incontinence or chronic pain, possibly because we did not manipulate the sphincters or anoderm. Ratto*et al.* ^*24*^ did not observe any alterations to faecal continence among their patients who underwent operations using the THD technique, from evaluations using anorectal manometry and endoanal ultrasound before the operation and six months afterwards. In a prospective study, comparing the THD technique with PPH (stapled haemorrhoidopexy), Giordano *et al.* ^*9*^ did not observe any alteration to faecal continence in the THD group, while two patients in the PPH group developed faecal urgency. In 2009, Khafagyv*et al* ^*15*^ published the results from clinical and functional evaluations (anorectal manometry) in a study comparing three operative techniques (conventional haemorrhoidectomy, PPH and THD), and concluded that, because the THD method was less invasive, it presented better functional results. Zampieri *et al.* ^35^ compared the long-term quality-of-life results after treatment of haemorrhoidal disease using the THD-M technique (in 46 patients) vs. haemorrhoidectomy with Ligasure (in 68 patients). In addition to a postoperative period with lower pain levels (p<0.05), they observed that functional results were better in the THD group because none of the patients experienced any alteration to faecal continence. On the other hand, in the Ligasure group, intestinal constipation with evacuation difficulty and greater need for analgesic and laxative medications were observed.

## CONCLUSION

The THD-M method is safe and effective for haemorrhoidal disease grades II and III with low rates of surgical complications. However, for grade IV hemorrhoids, it is associated with higher recurrence of prolapse and bleeding. So, THD-M method should not be considered as an effective option for the treatment of grade IV hemorrhoids.
